# Older persons are frailer after an emergency care visit to the out-of-hours general practitioner cooperative in the Netherlands: a cross-sectional descriptive TOPICS-MDS study

**DOI:** 10.1186/s12875-020-01220-y

**Published:** 2020-08-20

**Authors:** Anneke Bloemhoff, Yvonne Schoon, Kien Smulders, Reinier Akkermans, Lilian C. M. Vloet, Karin van den Berg, Sivera A. A. Berben, On behalf of the TOPICS-MDS Consortium

**Affiliations:** 1 Eastern Regional Emergency Healthcare Network, P.O. Box 9101, 6500 HB Nijmegen, The Netherlands; 2 Department of Emergency Medicine, Radboud University Medical Center, Nijmegen, The Netherlands; 3 Department of Geriatrics, Radboud University Medical Center, Nijmegen, The Netherlands; 4 General Practitioners Cooperative Gelderse Vallei, Ede, The Netherlands; 5 Radboud Institute for Health Sciences, IQ healthcare, Radboud University Medical Center, Nijmegen, The Netherlands; 6 Research Department of Emergency and Critical Care, HAN University of Applied Sciences, Nijmegen, The Netherlands

**Keywords:** Older persons, Frailty, General practitioners cooperative, Emergency care

## Abstract

**Background:**

In the Netherlands, community-dwelling older people with primary care emergency problems contact the General Practitioner Cooperative (GPC) after hours. However, frailty remains an often unobserved hazard with adverse health outcomes. The aim of this study was to provide insight into differences between older persons with or without GPC emergency care visits (reference group) regarding frailty and healthcare use.

**Methods:**

A cross-sectional descriptive study design was based on data from the public data repository of The Older Persons and Informal Caregivers Survey Minimum Dataset (TOPICS-MDS). Frailty in older persons (65+ years, *n* = 32,149) was measured by comorbidity, functional and psychosocial aspects, quality of life and a frailty index. Furthermore, home care use and hospital admissions of older persons were identified. We performed multilevel logistic and linear regression analyses. A random intercept model was utilised to test differences between groups, and adjustment factors (confounders) were used in the multilevel analysis.

**Results:**

Compared to the reference group, older persons with GPC contact were frailer in the domain of comorbidity (mean difference 0.52; 95% CI 0.47–0.57, *p* < 0.0001) and functional limitations (mean difference 0.53; 95% CI 0.46–0.60, *p* < 0.0001), and they reported less emotional wellbeing (mean difference − 4.10; 95% CI -4.59- -3.60, *p* < 0.0001) and experienced a lower quality of life (mean difference − 0.057; 95% CI -0.064- -0.050, *p* < 0.0001). Moreover, older persons more often reported limited social functioning (OR = 1.50; 95% CI 1.39–1.62, *p* < 0.0001) and limited perceived health (OR = 1.50, 95% CI 1.39–1.62, *p* < 0.0001). Finally, older persons with GPC contact more often used home care (OR = 1.37; 95% CI 1.28–1.47, *p* < 0.0001) or were more often admitted to the hospital (OR = 2.88; 95% CI 2.71–3.06, *p* < 0.0001).

**Conclusions:**

Older persons with out-of-hours GPC contact for an emergency care visit were significantly frailer in all domains and more likely to use home care or to be admitted to the hospital compared to the reference group. Potentially frail older persons seemed to require adequate identification of frailty and support (e.g., advanced care planning) both before and after a contact with the out-of-hours GPC.

## Background

Primary care settings that are open 24 h a day and 7 days a week vary within western countries in parallel to the different healthcare organization models in use [1, 2]. Out-of-hours primary care models are facing growing patient demands, an ageing population, increasing physician workloads and financial issues. In the Netherlands, the out-of-hours primary care model was changed around the year 2000 in response to these challenges. The current model in use employs large-scale general practitioner cooperatives (GPCs), in which 50–250 general practitioners take care of populations ranging from 100,000–500,000 citizens [3]. Today, most Dutch GPCs are co-located with emergency departments (EDs), where the GPC provides treatment to a large proportion of the patients who formerly presented to the ED [4].

Whereas worldwide an increasing demand for emergency care is perceived to be related to ageing [5, 6], in the Netherlands, one in six patients seen in an out-of-hours GPC is an older person (defined as 65+ years of age) [7]. Community-dwelling older people with emergency complaints often suffer from a variety of multiple, potentially life-threatening health conditions (e.g., cardiovascular, neurological, respiratory, musculoskeletal, abdominal, mental health conditions or a poor health status) [8]. The same study showed that repeated emergency healthcare visits (in the ED) were related to sociodemographic characteristics (socioeconomic status), social problems, health problems, need for a systematic health assessment, healthcare service use and inadequacy of previous or current care provided. However, data on revisits to the out-of-hours GPC were not found. Currently, the assessment of older persons in an out-of-hours GPC is mainly focused on physical problems and less or not at all focused on the assessment of the psycho-social domain of functioning. At the same time, the Longitudinal Ageing Study Amsterdam showed that older persons with an increased level of physical complaints often have increased levels of psychological problems as well [9].

An accumulation of increased physical, psychological and social limitations in daily functioning can be seen as a process of frailty [10]. There are at least 40 different operational definitions of frailty described in the geriatric medicine literature [11] varying from a more environmental [12] or cognitive perspective [12] to biopsychosocial models [13, 14]. This study used the biopsychosocial definition of frailty consistent with the measurement of frailty in the TOPICS-MDS studies [14]. The geriatric condition of frailty is characterised by an increased vulnerability to external stressors caused by the loss of reserve capacity in one or more domains of functioning [15–17]. However, the multifactorial process can be influenced in both a positive and a negative manner. Frailty in older persons is known to be related to a variety of adverse health outcomes, such as falls, functional decline, hospital admissions, and moreover, an increased risk of mortality [15, 16]. Therefore, it is important that frailty in older people is adequately addressed at an early stage in the (out-of-hours) primary care setting and that older persons are supported to prevent unnecessary adverse health outcomes. However, frailty of older persons in the out-of-hours GPC often remains undetected due to a lack of communication between the personal GP of the older person and the GPC. Furthermore, the physically oriented approach of GPCs for all patients, including older persons, results in a lack of identification of frailty due to other domain problems in older persons.

The hypothesis of this study was that older persons who visit the out-of-hours GPC with emergency complaints have an increased risk of being or becoming frail.

The aim of this study was to provide insights into the differences between older persons who contacted the out-of-hours GPC and a reference group of older persons without GPC contact regarding the level of frailty defined by co morbidity, functional limitations, psychosocial wellbeing, experienced quality of life, and healthcare use.

## Methods

### Design

In 2017, we performed a cross-sectional descriptive study based on data from The Older Persons and Informal Caregivers Survey Minimum Dataset (TOPICS-MDS) repository [18]. This public data repository contained information on physical and mental health, social wellbeing and health services utilization of older persons in the Netherlands. Projects with various study designs, sampling frames and inclusion criteria each delivered a minimal dataset based on standardised baseline measurements [14]. The included studies of the TOPICS-MDS repository were performed between 2009 and 2014. The TOPICS-MDS dataset for this study was fully anonymised, and therefore, our study was exempted from ethical review under Dutch law (reference number 2012/120) [14].

### Study population

**Fig. 1:**
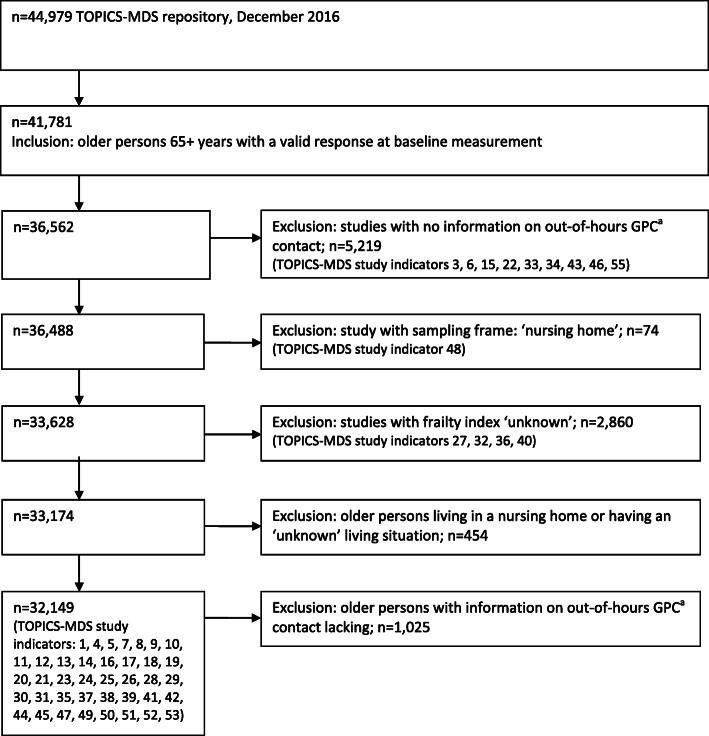
Flow chart of the study population. ^a^GPC = general practitioner cooperative

The TOPICS-MDS study population consisted of 55 studies containing a total of 44,979 older persons. We followed a stepwise inclusion and exclusion procedure at the study level, followed by an inclusion and exclusion procedure at the individual level to select the sample for our study (Fig. 1).

At study level, we included studies based on the following criteria:

1. The study focused on older persons (65+ years).
2. The sampling frame included older persons who depend on the GPC for out-of-hours primary care. In the Netherlands, this applies to community-dwelling older persons and to older people living in retirement homes. Older persons living in a nursing home were not included, because they receive institutionalised medical care from a specialised elderly care physician, who is never a primary care physician.
3. The study provided valid responses of older persons on baseline measurements.
4. The study included information of older persons regarding contact or absence of contact with the out-of-hours GPC.
5. The study included information on frailty of the older persons.

This procedure resulted in 39 studies with *n* = 33,628 respondents. At the individual level, we excluded respondents living in a nursing home, with an unknown living situation, and respondents with no valid data on GPC contact. The final study population consisted of *n* = 32,149 respondents (Fig. 1).

### Study variables

#### Demographic variables

Demographic variables in the study included gender, age, educational level, marital status, nationality, living situation and socioeconomic status of older persons. Age was classified into five categories: 65–69 years, 70–74 years, 75–79 years, 80–84 years, 85–89 years and 90 years and older. The educational level was classified as ‘low’, (defined as primary school or less education); ‘moderate’, (defined as 4-5 years of high school or vocational training); and ‘high’, (defined as university or tertiary education). Marital status was described as ‘married or living together’, ‘single or divorced’, or ‘widow (er)’. Nationality of the older persons was classified as either ‘Dutch’ or ‘first or second generation immigrants’. Living situation was described as ‘living at home’ or in a ‘retirement home’. The socioeconomic status (SES) sum score was based on income, employment and educational level and was determined at the postal code level in the Netherlands in 2010 [19]. In addition to the sum scores of the SES, separated information on income and (previous) employment of the older persons was not available through the TOPICS-MDS data repository. The SES score was classified into quartiles: ‘SES 1^st^ quartile’: -3.3 – -0.7; ‘SES 2^nd^ quartile’: -0.6 – 0.0; ‘SES 3^rd^ quartile’: 0.1–0.6; and ‘SES 4^th^ quartile’: 0.7–5.2, where higher SES scores represented a lower socioeconomic status of the older persons.

#### Frailty

The concept of frailty was operationalised according to the biopsychosocial definition of frailty and quantified by measurements in the original TOPICS-MDS studies through five corresponding domains (physical frailty, functional frailty, psychosocial frailty, quality of life and total frailty index) using seven indicators [14]:

- **Physical frailty:** the TOPICS-MDS baseline questionnaire included questions on comorbidities, including a total of 16 comorbidities of older persons (e.g., diabetes, heart failure, hip fracture). The sum comorbidity score (**CM)** ranged from 0 to 16, where higher CM scores represented more comorbidities and a higher level of physical frailty in older persons.
- **Functional frailty:** a modified version of the Katz Index of Independence Basic Activities of Daily Living (ADL), Instrumental Activities of Daily Living (IADL) and an additional indicator of mobility were used to quantify functional limitations of older persons in the TOPICS-MDS [20, 21]. The functional limitations score (**FL)** ranged from 0 to 15; higher scores represented more limitations and higher functional frailty in the older persons.
- **Psychosocial frailty**: The RAND-36 mental health subscale with five questions was used to assess emotional wellbeing (**EW**) [22, 23]. The EW score ranged from 0 to 100, where higher scores represented better emotional wellbeing and lower psychosocial frailty of older persons. Social functioning (**SF**) was also derived from a RAND-36 questionnaire. The response options of ‘all’ and ‘most of the time’ to the question, ‘how often in the past four weeks their physical health or emotional problems interfered with social activities’ indicated low SF and higher psychosocial frailty. The response options ‘fair’ and ‘poor’ to the question about their health in general were used to indicate a lower self-perceived health (**PH**) and higher psychosocial frailty.
- **Quality of life**: The TOPICS-MDS baseline questionnaire included the EuroQol Five Dimensions scale (**EQ-5D**) with application of the Dutch scoring values to measure the health-related quality of life [24, 25]. The EQ-5D utility score ranged from − 0.33 to 1.0; higher scores indicated a better quality of life in older persons.
- **Frailty index**: Finally, a more comprehensive composite frailty measurement was used: the long TOPICS-MDS frailty index (**FI**), based on the concept of deficit accumulation. This index consists of 46 items, all included in the TOPICS-MDS baseline questionnaire and related to the physical, functional and psychosocial domains [26]. In this study, the frailty index ranged from 0.0–0.85, where a higher level of frailty of older persons is indicated by a higher score on the FI.

#### GPC contact and healthcare use

To differentiate between older persons with and without GPC contact, the following question in the TOPICS-MDS baseline questionnaire was used: ‘Did you visit the general practitioner or did the general practitioner visit you during evenings, nights or weekends over the last 12 months? The response options were either ‘Yes’ or ‘No’.

The variable ‘healthcare use’ was defined as ‘hospital admission’ and ‘use of home care’. For hospital admission the question in the TOPICS-MDS baseline questionnaire was used: ‘Have you been hospitalised in the last 12 months?’ The response options were either ‘Yes’ or ‘No’. For home care use the question in the TOPICS-MDS baseline questionnaire was used: ‘Have you used home care?’. In the Netherlands, the definition of home care comprehends formal home care nursing and/or domestic help paid by the local government or the health insurance company. The response options in the questionnaire were either ‘Yes’ or ‘No’.

### Key outcome measures

The key outcome measurement of the study was frailty of older persons in the five domains presented earlier. The secondary outcome measurement of the study concerned the healthcare use of older persons, defined as hospital admission and or the older persons’ use of home care.

The independent variable of the study was contact of the older person with the out-of-hours GPC.

### Statistical analysis

Respondents with and without GPC contact were compared with respect to demographic characteristics, frailty and healthcare use. Because of the hierarchical structure of our study (patient nested within studies), we performed multilevel (mixed model) analyses. We performed multilevel logistic regression analysis for dichotomous outcome measures and multilevel linear regressions analysis for continuous outcome measures. A random intercept model was used to test the difference between the group with GPC contact and the group with no GPC contact. Demographic variables that significantly differed between the group of older persons with GPC contact and the group of persons with no GPC contact were used as adjustment factors (confounders) in the multilevel analysis for outcome measures. Furthermore, we adjusted for SES in our frailty analyses, because frailty is associated with socioeconomic inequalities [27, 28]. Finally, we adjusted for hospital admission in the analysis of frailty in older persons, as this appeared to be a significant relevant factor. A *p*-value of < 0.05 was considered to be statistically significant based on two-sided tests. We analysed the data by using the statistical software programme SPSS version 22.

## Results

In total, 32,149 older persons were included in the study from 39 different studies in the TOPICS-MDS repository; see Table 1.

**Table 1:** Study population by TOPICS-MDS study indicator, number of respondents and percentage (*n* = 32,149)

Study indicator	older persons n	%	Study indicator	older persons n	%
1	1769	5.5	26	105	0.3
4	2418	7.5	28	1466	4.6
5	2256	7.0	29	16	0.0
7	828	2.6	30	60	0.2
8	1113	3.5	31	170	0.5
9	978	3.0	35	213	0.7
10	3131	9.7	37	75	0.2
11	148	0.5	38	371	1.2
12	1539	4.8	39	91	0.3
13	574	1.8	41	491	1.5
14	556	1.7	42	206	0.6
16	1140	3.5	44	247	0.8
17	124	0.4	45	52	0.2
18	6391	19.9	47	43	0.1
19	444	1.4	49	120	0.4
20	46	0.1	50	469	1.5
21	915	2.8	51	1012	3.1
23	473	1.5	52	332	1.0
24	406	1.3	53	152	0.5
25	1209	3.8	**Total**	**32,149**	**100.0**

Nearly a quarter of the older persons (*n* = 7647; 23.8%) reported having had contact with the GPC, through either an out-of-hours GP consult at the GPC or a GP home visit. The demographic characteristics of the older persons with and without out-of-hours contact with the GPC are presented in Table 2.

**Table 2:** Demographic characteristics of older persons with and without out-of-hours GPC^a^ contact (*n* = 32,149)

Characteristics (n)	out-of-hours GPC contact		no out-of-hours GPC contact		OR^b^	95% CI^c^	*P*-value^d^
	n	%	n	%			
*Gender (32,136)*							**< 0.0001**
Male	3239	25.2	9601	74.8	1.13	1.07–1.20	
Female	4402	22.8	14,894	77.2	1		
*Age (32,149)*							**< 0.0001**
90 years and older	598	21.8	2141	78.2	1.11	0.98–1.25	
85–89 years	1318	25.2	3909	74.8	1.27	1.16–1.40	
80–84 years	1882	24.0	5955	76.0	1.16	1.07–1.27	
75–79 years	1870	22.2	6566	77.8	1.05	0.97–1.15	
70–74 years	1339	22.4	4643	77.6	1		
65–69 years	640	33.2	1288	66.8	1.10	0.97–1.24	
*Educational level (31,883)*							**0.017**
Low	2802	25.2	8306	74.8	1.09	1.03–1.16	
Moderate	3531	23.1	11,747	76.9	1		
High	1248	22.7	4249	77.3	1.02	0.94–1.10	
*Marital status (32,083)*							0.876
Widow (er)	2939	23.3	9672	76.7	0.99	0.93–1.05	
Divorced/Single	879	24.5	2709	75.5	0.98	0.90–1.07	
Married/Living together	3804	23.9	12,080	76.1	1		
*Nationality (31,708)*							0.439
First/second generation immigrants	770	27.5	2035	72.5	1.04	0.94–1.14	
Dutch	6782	23.5	22,121	76.5	1		
*Living situation (32,149)*							0.250
Retirement home	734	21.0	2754	79.0	1.07	0.95–1.21	
At home	6913	24.1	21,748	75.9	1		
*SES*^*e*^ *(30,118)*							**0.008**
SES 4th quartile (low SES)	1940	26.5	5381	73.5	1.14	1.05–1.25	
SES 3rd quartile	1621	24.8	4921	75.2	1.14	1.04–1.24	
SES 2nd quartile	2085	22.9	7022	77.1	1.13	1.04–1.22	
SES 1st quartile (high SES)	1424	19.9	5724	80.1	1		

^a^ *GPC* general practitioner cooperative. ^b^ *OR* Odds Ratio based on multilevel analysis, with correction for cluster effects between studies. ^c^ *CI* confidence interval. ^d^ bold *p*-values are statistically significant. ^e^ *SES* socioeconomic status

Males were significantly more likely to contact the GPC than women (OR = 1.13; 95% CI 1.07–1.20). Furthermore, the age of the older persons (categorised by 5 year clusters) was also shown to be significantly related (*p* < 0.0001) to GPC contact. Older persons between 80 and 90 years of age were most likely to have had GPC contact. The risk of GPC contact differed significantly by educational levels (*p* = 0.017) and SES-levels (*p* = 0.008) of the older persons; persons with a low educational level had the highest risk, and persons with a high SES had the lowest risk of contacting the GPC (Table 2). Other demographic variables showed slight differences between older persons with or without GPC contact, although these differences were not statistically significant.

The use of healthcare was measured by hospital admission in the last 12 months and use of home care. A quarter of the older persons (*n* = 8190; 25.7%) reported being admitted to the hospital and a third of the older persons (*n* = 10,118; 36%) reported to have received home care. The association between GPC contact and the use of professional healthcare is presented in Table 3. Older persons who had GPC contact were more likely to be admitted to the hospital (OR = 2.88; 95% CI 2.71–3.06) or to use home care (OR = 1.37; 95% CI 1.28–1.47) compared to older persons who had no GPC contact.

**Table 3:** Healthcare use characteristics of older persons with and without out-of-hours GPC^a^ contact (*n* = 32,149)

Use of professional healthcare (n)	out-of-hours GPC contact		no out-of-hours GPC contact		OR^b^	95% CI^c^	*P*-value^d^
	n	%	n	%			
*Admission to the hospital (29,824)*							**< 0.0001**
Yes	3204	39.1	4986	60.9	2.88	2.71–3.06	
No	4343	18.3	19,334	81.7	1		
*Home care (31,602)*^e^							**< 0.0001**
Yes	3152	28.8	7811	71.2	1.37	1.28–1.47	
No	4294	20.8	16,345	79.2	1		

^a^ *GPC* general practitioner cooperative. ^b^ *OR* Odds Ratio based on multilevel analysis, adjusted for gender, age, socioeconomic status and cluster effects between studies. ^c^ *CI* confidence interval. ^d^ bold p-values are statistically significant. ^e^ only for older persons living at home

Thirty-nine percent (*n* = 12,384) of the older persons were found to be frail by scoring 0.25 or higher as measured with the TOPICS-MDS frailty index. The associations between GPC contact and different domains of the concept of frailty in older persons are presented in Table 4. Overall, older persons with GPC contact were significantly more frail compared to older persons with no GPC contact, and these differences were apparent in all domains (*p* < 0.0001). Older persons with GPC contact had more comorbidities and were more often limited in daily activities and social functioning. The quality of life experience and psychosocial wellbeing of older persons with GPC contact were significantly lower compared to older persons who had no GPC contact.

**Table 4:** Association between frailty and (no) out-of-hours GPC^a^ contact of older persons (*n* = 32,149)

**Indicators of frailty**	**older persons n**	**out-of-hours GPC**^**a**^ **contact mean**	**no out-of-hours GPC**^**a**^ **contact mean**	**difference**^**b**^	**95% CI**^**c**^	***P*-value**^**d**^
Frailty index TOPICS-MDS (FI)^e^	29,465	0.27	0.21	0.037	0.034–0.040	**< 0.0001**
Comorbidity (CM)^f^	29,672	3.43	2.76	0.52	0.47–0.57	**< 0.0001**
Functional limitations (FL)^g^	29,759	3.57	2.82	0.53	0.46–0.60	**< 0.0001**
Emotional wellbeing (EW)^h^	29,334	69.69	74.95	-4.10	-4.59 - -3.60	**< 0.0001**
Quality of life (EQ-5D)^i^	29,287	0.65	0.73	-0.057	-0.064 - -0.050	**< 0.0001**
**Indicators of frailty**	**older persons n**	**out-of-hours GPC**^**a**^ **contact %**	**no out-of-hours GPC**^**a**^ **contact %**	**OR**^**j**^	**95% CI**^**c**^	***p*-value**^**d**^
Limited social functioning (SF)	28,993	23.8	13.8	1.50	1.39–1.62	**< 0.0001**
Limited perceived health (PH)	29,728	57.8	42.3	1.61	1.51–1.71	**< 0.0001**

^a^ *GPC* general practitioner cooperative. ^b^ Difference between out-of-hours GPC contact and no out-of-hours GPC contact. ^c^ *CI* confidence interval. ^d^ bold p-values are statistically significant. ^e^ sum score range 0.00–0.85; a higher score represents a higher level of frailty. ^f^ sum score range 0–16; a higher score represents more reported comorbidities. ^g^ sum score range 0–15; a higher score represents more functional limitations in daily living activities. ^h^ sum score range 0–100; a higher score represents more emotional wellbeing. ^i^ sum score range − 0.33 – 1.00; a higher score represents a better quality of life. ^j^ OR = Odds Ratio based on multilevel analysis with correction for gender, age, socioeconomic status, hospital admission and cluster effects between studies

## Discussion

### Principal findings

This secondary analysis of the TOPICS-MDS data provided insights into differences in the level of frailty and healthcare use of older persons with or without out-of-hours GPC contact. This study showed that persons of 65 years and older who had contact with the GPC and received either an emergency consult at the GPC or an urgent GP home visit were significantly more frail compared to older persons who had no GPC contact during out-of-office hours. Furthermore, we observed an increased level of frailty in all domains (e.g., comorbidity, activities of daily living, social contacts, psychological wellbeing, and quality of life). In addition, older persons who visited the GPC were significantly more often admitted to the hospital and more often used home care compared to older persons who had no contact with the GPC. In summary, this study shows that older people who had contact with the GPC had a relevant problem with frailty. Previous studies on frailty of older persons receiving emergency care have usually focused on the setting of the emergency department [29–32] or the general practitioners’ practice during daytime hours [33–36]. Studies in the last group did not differentiate between regular, chronic and emergency primary healthcare. To our knowledge, this is the first study that identifies the frailty of older persons after contact with the emergency primary healthcare setting during out-of-hours.

The identified differences between older persons with GPC contact and the reference group, all of which supported the findings. We showed statistically significantly increased levels of frailty in older persons who contacted the GPC. However, it is unclear whether the identified differences were clinically relevant for older persons. As the differences in frailty levels of older persons with (out) contact with the GPC were shown to be (more than) 5%, we also considered the identified levels of frailty to be clinically relevant [37].

This secondary data analysis was based on TOPICS-MDS data retrieved from the baseline measurements in studies that were performed in the period between 2009 and 2014. Since that time, several policy measures have been undertaken to reform the Dutch healthcare system, such as a reduction or abolishment of retirement homes and the introduction of increased thresholds for admission of older persons to nursing homes. Additionally, the financial reimbursement systems for long term care facilities (e.g., for older persons) changed in the Netherlands. As a result, more older people now remain at home longer with an accumulation of comorbidities, psychosocial problems, and at the same time a (temporary) loss of reserve capacity in one or more domains of functioning. Accumulation of frailty in community-dwelling older persons leads to a crisis where older people seek emergency (primary) healthcare. Additionally, in the (inter) national literature, the problem of emergency department crowding related to ageing [5, 38] and an increased number of vulnerable older persons requiring care by general practitioners [7] has been described. The effects of the earlier described healthcare system reform measures were not represented in the TOPICS-MDS database in the period between 2009 and 2014. Therefore, our findings could underestimate the actual problem of frailty in community-dwelling older persons who currently seek emergency healthcare.

We know that access to and use of (primary) healthcare is highly influenced by SES, and this study also showed a positive association between a low SES and higher GPC use. Furthermore, Shebehe et al. 2018 showed that older persons above 65 years of age who visited primary health care centres in Sweden localised in neighbourhoods with a low SES had higher rates of hospital readmission compared to older persons with a higher SES [39]. This result suggests that interventions aimed at empowering older persons, reducing frailty in primary care, and reducing hospital readmissions for older persons should also take socioeconomic disparities into consideration.

Older persons rarely use the word ‘frail’ to describe their situation, because they do not think about themselves in terms of frailty [40]. Older persons who are classified as frail according to medical criteria do not always feel frail [41, 42]. This difference in interpretation between doctors and patients is also known as the ‘disability paradox’ [43]. The discrepancy between the clinical understanding of frailty and the way people perceive frailty has important implications for older people’s wellbeing [44]. While frailty classifications can be useful in guiding clinical care, it is also important to consider how individuals and others perceive and respond to their experiences of frailty. Older persons described that being labelled as frail – particularly against one’s will – was seen as damaging to their health, because it may lead to behavioural confirmation of the label. When labelling occurs, unless older persons possess strategies to resist this trend, their health and wellbeing may be compromised. Frail older persons are mainly concerned with their quality of life, asking questions such as ‘what is important’, ‘what do I value’ and ‘what gives meaning to my life’. At the same time, older persons consider it relevant to talk to healthcare professionals about aspects of physical decline, psychosocial reserve capacities and (emergency) primary healthcare treatment in terms of opportunities and threats (risks) [40]. As frailty is a process involving an accumulation of physical, psychological and/or social deficits in functioning with the risk of adverse health outcomes (admission to a hospital or sometimes death) [45], advanced care planning between older persons and their GP is important [46]. Furthermore, circumstances and preferences in the health care of older persons can rapidly change because of the occurrence of (adverse) events, such as a crisis or a hospital admission [47]. The preference of older persons to what extent and how they prefer to be involved in shared decision making in primary healthcare varies [48], whereas GP characteristics, communication skills, GP consultation duration, and continuity of (emergency) healthcare were described as important factors in the enhancement of shared decision making [49, 50]. GPs themselves showed different perspectives on their role in the management of complex health problems of older persons in primary care, varying from ‘manoeuvring along competence limits’, ‘Herculean task’, and ‘cooperation and networking’ [51].

In our opinion, the primary care GP plays an important role in the early identification of frailty, advanced care planning and the management of complex interventions in older persons. Where more adequate support of frail older persons can be provided, possibly (unnecessary) out-of-hours GPC contacts or ED admissions can be avoided. Brouwers et al. 2017 showed in an explorative study amongst emergency healthcare providers that early identification of frailty, improving the continuity between primary and home healthcare and hospital based (emergency) care and vice versa is recommended [52]. In recent years, several instruments for the screening of frailty in older persons have been developed. Many of these instruments focus on the identification of physical, functional or cognitive aspects of frailty [34, 53], such as the ISAR (Identification of Seniors at Risk) [32], the SHARE-FI (Survey of Health, Ageing and Retirement in Europe Frailty Instrument) [31], and the APOP screener [54], while other instruments are focused on a multi-dimensional screening of frailty in the elderly such as the Groningen Frailty Indicator [55], the Frail-VIG [53] and Easy-Care TOS [33, 56]. Based on our study results, we recommend developing building blocks and tools for a multi-dimensional screening of frailty in emergency primary healthcare settings such as the GPC. Early detection of frailty in primary care can improve the use of effective interventions in primary care that provide adequate biopsychosocial support in older persons and thus prevent frailty progression [57]. A recent systematic review showed that group sessions, individual educational sessions by a geriatrician and cognitive training for older persons in primary care had a positive effect on improvements in frailty, physical activity and other outcomes [57]. In addition to early detection and effective (preventive) interventions, follow-up care for older persons after a GPC visit also seems important.

If accessibility of older persons to primary care (including out-of-hours primary care) is high, than the rate of ED visits is significantly lower [58]. Furthermore, structured and structural information exchange between healthcare providers in the emergency healthcare pathway and a more generalist approach of older persons in emergency healthcare is recommended in order to deliver appropriate emergency healthcare for older persons who are frail.

### Strengths and limitations

An important strength of the TOPICS-MDS study is the large sample and therefore the ecological validity of the study results. Furthermore, differences between older persons who have (or have not) had contact with the GPC were shown to be statistically and clinically significant. However, the study sample contains a combination of studies with different designs, inclusion and exclusion criteria, sample sizes, and data collection methods. Therefore, the representativeness of this cross-sectional study sample for the whole Dutch population of persons 65 years or older could be questioned. Nevertheless, our results and associations are not less relevant [59]. Furthermore, we corrected for the potential confounding demographic variables (gender, age and socioeconomic status) and for cluster effects between studies in the multilevel statistical analysis.

A disadvantage of secondary data analysis is that data quality control is challenging. For instance, for the ‘healthcare use’ outcome measures, we have chosen to include only information on hospital admissions and the use of home care in the analysis, and we omitted temporary admission of older persons to a retirement or nursing home as an outcome measurement due to the insufficient quality of these data.

The data analysis was based on baseline measurements of older persons after they had GPC contact. Therefore, it is unclear whether frailty was present before the GPC contact. It is possible that frailty predicts a GPC contact of older persons, or perhaps a visit of an older person to the GPC results in increasing levels of frailty. Additionally, the cross-sectional study design could be considered a limitation.

Finally, the assumption that data from the primary studies included in this secondary data analysis are robust is an important point of departure in the multilevel analysis. However, during cross data quality checks, data of some variables appeared to be incorrect, such as the date and time of the measurement at T0. Where possible, we corrected these data based on the original study publications. Despite these efforts, we cannot completely rule out some misclassifications.

## Conclusions

Older persons who had out-of-hours GPC contact were significantly frailer in all domains (comorbidity, functional limitations, psychosocial wellbeing, and experienced quality of life) compared to older persons without out-of-hours GPC contact. Furthermore, they were more likely to be admitted to the hospital or to use home care compared to older persons who had no GPC contact. These findings imply that frailty in older persons should be identified early in primary care, either by their own GP or at the GPC, in order to organize advanced care planning and to possibly prevent admission to the out-of-hours GPC. Moreover, adequate follow-up and support of older persons after a contact with the out-of-hours GPC requires (more) structural attention.

## Data Availability

The TOPICS-MDS data repository has been archived as a thematic collection by the TOPICS-MDS Project Group in DANS, the Dutch institute for permanent access to digital research resources. DOI: doi.org/10.17026/dans-xvh-dbbf. The thematic data collection comprehends three different datasets: 1) care receiver, 2) care giver and 3) care receiver – care giver dyads. The data selection used for this study originated from the first data set, doi: doi.org/10.17026/dans-xwf-g759. The TOPICS-MDS data are available through restricted access and after approval of the TOPICS-MDS Project Group. All other documents (background information on TOPICS-MDS, questionnaires, codebooks, SPSS syntax and metadata) are open access available.

## Abbreviations

ADL: Activities of Daily Living
APOP: Acutely Presenting Older Patients
CI: Confidence Interval
CM: Co-Morbidity
Easy-Care TOS: European Assessment SYstem-Care Two-stage Ouderen (Elderly) Screening
ED: Emergency Department
EQ-5D: EuroQol Five Dimensions scale
EW: Emotional Wellbeing
FI: Frailty Index
FL: Functional Limitations
Frail-VIG: VIG is the Spanish/Catalan abbreviation for comprehensive geriatric assessment
GP: General Practitioner
GPC: General Practitioner Cooperative
IADL: Instrumental Activities of Daily Living
ISAR: Identification of Seniors at Risk
OR: Odds Ratio
PH: Self-Perceived Health
SES: SocioEconomic Status
SF: Social Functioning
SHARE-FI: Survey of Health, Ageing and Retirement in Europe Frailty Instrument
TOPICS-MDS: The Older Persons and Informal Caregivers Survey Minimum Data Set

## References

[1] Huibers L, Giesen P, Wensing M, Grol R (2009). Out-of-hours care in western countries: assessment of different organizational models. BMC Health Serv Res.

[2] Leibowitz R, Day S, Dunt D (2003). A systematic review of the effect of different models of after-hours primary medical care services on clinical outcome, medical workload, and patient and GP satisfaction. Fam Pract.

[3] Smits M, Rutten M, Keizer E, Wensing M, Westert G, Giesen P (2017). The development and performance of after-hours primary Care in the Netherlands: a narrative review. Ann Intern Med.

[4] Rutten M, Vrielink F, Smits M, Giesen P (2017). Patient and care characteristics of self-referrals treated by the general practitioner cooperative at emergency-care-access-points in the Netherlands. BMC Fam Pract.

[5] Lowthian JA, Jolley DJ, Curtis AJ, Currell A, Cameron PA, Stoelwinder JU (2011). The challenges of population ageing: accelerating demand for emergency ambulance services by older patients, 1995-2015. Med J Aust.

[6] Kisser R, Walters A, Rogmans W, Turner S, Lyons RA. Injuries in the European Union 2013-2015. Amsterdam: European Associaton for Injury Prevention and Safety Promotion (EuroSafe); 2017. https://www.eurosafe.eu.com/uploads/inline-files/IDB%202013-2015_suppl%20to%206th%20edition%20Injuries%20in%20the%20EU.pdf. Accessed Jan 2020.

[7] Huibers L, Moth G, Andersen M, van Grunsven P, Giesen P, Christensen MB (2014). Consumption in out-of-hours health care: Danes double Dutch?. Scand J Prim Health Care.

[8] Steinmiller J, Routasalo P, Suominen T (2015). Older people in the emergency department: a literature review. Int J Older People Nursing.

[9] Hoogendijk EO, van Hout HP, van der Horst HE, Frijters DH, Dent E, Deeg DJ (2014). Do psychosocial resources modify the effects of frailty on functional decline and mortality?. J Psychosom Res.

[10] Hoogendijk EO, Theou O, Rockwood K, Onwuteaka-Philipsen BD, Deeg DJ, Huisman M (2016). Development and validation of a frailty index in the longitudinal aging study Amsterdam. Aging Clin Exp Res.

[11] Cesari M, Calvani R, Marzetti E (2017). Frailty in older persons. Clin Geriatr Med.

[12] Freer K, Wallington SL (2019). Social frailty: the importance of social and environmental factors in predicting frailty in older adults. Br J Community Nurs.

[13] Gobbens RJ, Luijkx KG, Wijnen-Sponselee MT, Schols JM (2010). In search of an integral conceptual definition of frailty: opinions of experts. J Am Med Dir Assoc.

[14] Lutomski JE, Baars MA, Schalk BW, Boter H, Buurman BM, den Elzen WP (2013). The development of the older persons and informal caregivers survey minimum DataSet (TOPICS-MDS): a large-scale data sharing initiative. PLoS One.

[15] Clegg A, Young J, Iliffe S, Olde Rikkert M, Rockwood K (2013). Frailty in elderly people. Lancet..

[16] Fried LP, Tangen CM, Walston J, Newman AB, Hirsch C, Gottdiener J (2001). Frailty in older adults: evidence for a phenotype. J Gerontol A Biol Sci Med Sci.

[17] Morley JE, Vellas B, van Kan GA, Anker SD, Bauer JM, Bernabei R (2013). Frailty consensus: a call to action. J Am Med Dir Assoc.

[18] The Older Persons and Informal Caregivers Survey Minimum Dataset (TOPICS-MDS). https://topics-mds.eu/. [Accessed December 2019].

[19] Sociaal Cultureel Planbureau. Statusscores 2018 [updated 14-06-2018]. https://www.scp.nl/Onderzoek/Lopend_onderzoek/A_Z_alle_lopende_onderzoeken/Statusscores [Accessed January 2019].

[20] Weinberger M, Samsa GP, Schmader K, Greenberg SM, Carr DB, Wildman DS (1992). Comparing proxy and patients’ perceptions of patients’ functional status: results from an outpatient geriatric clinic. J Am Geriatr Soc.

[21] Lawton MP, Brody EM (1969). Assessment of older people: self-maintaining and instrumental activities of daily living. Gerontologist..

[22] Aaronson NK, Muller M, Cohen PD, Essink-Bot ML, Fekkes M, Sanderman R (1998). Translation, validation, and norming of the Dutch language version of the SF-36 health survey in community and chronic disease populations. J Clin Epidemiol.

[23] RAND Corporation. 36-Item Short Form Survey (SF-36). https://www.rand.org/health-care/surveys_tools/mos/36-item-short-form.html. [Accessed December 2019].

[24] EuroQol. EQ-5D-3L [updated 18-04-2017]. https://euroqol.org/eq-5d-instruments/eq-5d-3l-about/. [Accessed December 2019].

[25] Lamers LM, Stalmeier PF, McDonnell J, Krabbe PF, van Busschbach JJ (2005). Measuring the quality of life in economic evaluations: the Dutch EQ-5D tariff. Ned Tijdschr Geneeskd.

[26] Lutomski JE, Baars MA, van Kempen JA, Buurman BM, den Elzen WP, Jansen AP (2013). Validation of a frailty index from the older persons and informal caregivers survey minimum data set. J Am Geriatr Soc.

[27] Herr M, Robine JM, Aegerter P, Arvieu JJ, Ankri J (2015). Contribution of socioeconomic position over life to frailty differences in old age: comparison of life-course models in a French sample of 2350 old people. Ann Epidemiol.

[28] Brunner EJ, Shipley MJ, Ahmadi-Abhari S, Valencia Hernandez C, Abell JG, Singh-Manoux A (2018). Midlife contributors to socioeconomic differences in frailty during later life: a prospective cohort study. Lancet Public Health.

[29] Thiem U, Heppner HJ, Singler K (2015). Instruments to identify elderly patients in the emergency department in need of geriatric care. Z Gerontol Geriatr.

[30] Suffoletto B, Miller T, Shah R, Callaway C, Yealy DM (2016). Predicting older adults who return to the hospital or die within 30 days of emergency department care using the ISAR tool: subjective versus objective risk factors. Emerg Med J.

[31] Fallon A, Kilbane L, Briggs R, Dyer A, Nabeel S, McElwaine P (2018). Screening for frailty in older emergency department patients: the utility of the survey of health, ageing and retirement in Europe frailty instrument (SHARE-FI). QJM..

[32] Yao JL, Fang J, Lou QQ, Anderson RM (2015). A systematic review of the identification of seniors at risk (ISAR) tool for the prediction of adverse outcome in elderly patients seen in the emergency department. Int J Clin Exp Med.

[33] van Kempen JA, Schers HJ, Melis RJ, Olde Rikkert MG (2014). Construct validity and reliability of a two-step tool for the identification of frail older people in primary care. J Clin Epidemiol.

[34] Sutorius FL, Hoogendijk EO, Prins BA, van Hout HP (2016). Comparison of 10 single and stepped methods to identify frail older persons in primary care: diagnostic and prognostic accuracy. BMC Fam Pract.

[35] van Kempen JA, Schers HJ, Philp I, Olde Rikkert MG, Melis RJ (2015). Predictive validity of a two-step tool to map frailty in primary care. BMC Med.

[36] Romero-Ortuno R (2015). Frailty in primary care. Interdiscip Top Gerontol Geriatr.

[37] Eendebak R, Theou O, van der Valk A, Godin J, Andrew M, McNeil S (2018). Defining minimal important differences and establishing categories for the frailty index. Innov Aging.

[38] Lowthian J, Curtis A, Stoelwinder J, McNeil J, Cameron P (2013). Emergency demand and repeat attendances by older patients. Intern Med J.

[39] Shebehe J, Hansson A (2018). High hospital readmission rates for patients aged >/=65 years associated with low socioeconomic status in a Swedish region: a cross-sectional study in primary care. Scand J Prim Health Care.

[40] Verhoeven A, Kooiker S, van Campen C (2011). Frail older persons in the Netherlands. Perspectives of older persons regarding frailty and quality of life.

[41] von Faber M (2002). Maten van succes bij ouderen. Gezondheid, aanpassing en sociaal welbevinden (in Dutch).

[42] Puts MT, Shekary N, Widdershoven G, Heldens J, Lips P, Deeg DJ (2007). What does quality of life mean to older frail and non-frail community-dwelling adults in the Netherlands?. Qual Life Res.

[43] Albrecht GL, Devlieger PJ (1999). The disability paradox: high quality of life against all odds. Soc Sci Med.

[44] Warmoth K, Lang IA, Phoenix C, Abraham C, Andrew MK, Hubbards RE (2016). Thinking you're old and frail’: a qualitative study of frailty in older adults. Aging Soc.

[45] Berben SAA, Bloemhoff A, Habets KCF, Liefers J, Hensens CJM, van Grunsven PM (2019). Care contacts of elderly patients in the emergency care pathway: a retrospective cohort study. Ned Tijdschr Geneeskd.

[46] Suen LW, Leyde S, Min K, Volow A, Rabow M, Sudore RL (2019). Thinking outside the visit: primary care patient perspectives on helpful advanced care planning methods. J Gen Intern Med.

[47] Muth C, van den Akker M, Blom JW, Mallen CD, Rochon J, Schellevis FG (2014). The Ariadne principles: how to handle multimorbidity in primary care consultations. BMC Med.

[48] Chi WC, Wolff J, Greer R, Dy S (2017). Multimorbidity and decision-making preferences among older adults. Ann Fam Med.

[49] Butterworth JE, Campbell JL (2014). Older patients and their GPs: shared decision making in enhancing trust. Br J Gen Pract.

[50] Hoffmann T, Jansen J, Glasziou P (2018). The importance and challenges of shared decision making in older people with multimorbidity. PLoS Med.

[51] Herzog A, Gaertner B, Scheidt-Nave C, Holzhausen M (2015). We can do only what we have the means for’ general practitioners’ views of primary care for older people with complex health problems. BMC Fam Pract.

[52] Brouwers C, Merten H, Willems M, Habraken DJ, Bloemers FW, Biesheuvel TH (2017). Improving care for older patients in the acute setting: a qualitative study with healthcare providers. Neth J Med.

[53] Amblas-Novellas J, Martori JC, Espaulella J, Oller R, Molist-Brunet N, Inzitari M (2018). Frail-VIG index: a concise frailty evaluation tool for rapid geriatric assessment. BMC Geriatr.

[54] de Gelder J, Lucke JA, Blomaard LC, Booijen AM, Fogteloo AJ, Anten S (2018). Optimization of the APOP screener to predict functional decline or mortality in older emergency department patients: cross-validation in four prospective cohorts. Exp Gerontol.

[55] Peters LL, Boter H, Buskens E, Slaets JP (2012). Measurement properties of the Groningen frailty indicator in home-dwelling and institutionalized elderly people. J Am Med Dir Assoc.

[56] van Kempen JA, Schers HJ, Jacobs A, Zuidema SU, Ruikes F, Robben SH (2013). Development of an instrument for the identification of frail older people as a target population for integrated care. Br J Gen Pract.

[57] Apostolo J, Cooke R, Bobrowicz-Campos E, Santana S, Marcucci M (2018). Effectiveness of interventions to prevent pre-frailty and frailty progression in older adults: a systematic review. JBI Database System Rev Implement Rep.

[58] Or Z, Penneau A (2018). A multilevel analysis of the determinants of emergency care visits by the elderly in France. Health Policy.

[59] Rothman KJ, Gallacher JE, Hatch EE (2013). Why representativeness should be avoided. Int J Epidemiol.

